# Improved screening of retinopathy of prematurity (ROP): development of a target product profile (TPP) for resource-limited settings

**DOI:** 10.1136/bmjophth-2022-001197

**Published:** 2023-07-17

**Authors:** Rebecca P Kirby, Aeesha N J Malik, Kara M Palamountain, Clare E Gilbert, TPP Survey and Meeting Participants Collaborator Group, Afsar Dastjani Farahani

**Affiliations:** 1 Kellogg School of Management, Northwestern University, Evanston, Illinois, USA; 2 Department of Clinical Research, London School of Hygiene & Tropical Medicine, London, UK

**Keywords:** child health (paediatrics), public health

## Abstract

**Background:**

As more preterm infants survive, complications of preterm birth, including retinopathy of prematurity (ROP), become more prevalent. ROP rates and blindness from ROP are higher in low-income and middle-income countries, where exposure to risk factors can be higher and where detection and treatment of ROP are under-resourced or non-existent. Access to low-cost imaging devices would improve remote screening capabilities for ROP.

**Methods:**

Target product profiles (TPPs) are developed early in the medical device development process to define the setting, target user and range of product requirements. A Delphi-like process, consisting of an online survey and consensus meeting, was used to develop a TPP for an ROP imaging device, collecting feedback on a proposed set of 64 product requirements.

**Results:**

Thirty-six stakeholders from 17 countries provided feedback: clinicians (72%), product developers (14%), technicians (6%) and other (8%). Thirty-six per cent reported not currently screening for ROP, with cited barriers including cost (44%), no training (17%) and poor image quality (16%). Among those screening (n=23), 48% use more than one device, with the most common being an indirect ophthalmoscope (87%), followed by RetCam (26%) and smartphone with image capture (26%). Consensus was reached on 53 (83%) product requirements. The 11 remaining were discussed at the consensus meeting, and all but two achieved consensus.

**Conclusions:**

This TPP process was novel in that it successfully brought together diverse stakeholders to reach consensus on the product requirements for an ROP imaging devices. The resulting TPP provides a framework from which innovators can develop prototypes.

**WHAT IS ALREADY KNOWN ON THIS TOPIC**

- More preterm infants are now surviving, and a larger number are developing complications of preterm birth, including retinopathy of prematurity (ROP); however, there is a lack of affordable screening technologies available in low-income and middle-income countries.
- As a result, access to these medical devices remains a barrier in low-income and middle-income countries.

**WHAT THIS STUDY ADDS**

- NEST360, the London School of Hygiene & Tropical Medicine and UNICEF led a Delphi-like survey process and consensus meeting with global experts to define the product requirements for a target product profile (TPP) for an imaging device for ROP screening.
- The online survey included responses from 36 stakeholders across 17 countries and 56 stakeholders attended the consensus meeting resulting in a TPP that defines the characteristics of an easy-to-use, low-cost imaging device which can be used to screen for ROP in low-income and middle-income countries.

**HOW THIS STUDY MIGHT AFFECT RESEARCH, PRACTICE OR POLICY**

- The process adopted in developing the TPP successfully brought together diverse stakeholders to reprioritise an increasingly important complication of preterm birth.
- The resulting TPP provides a framework which innovators can refer to while developing prototypes that lead to new technologies for an imaging device for ROP screening.

## Introduction

The neonatal period (ie, the first 28 days of life) is the most vulnerable time for a child’s survival. As advances in the ability to resuscitate and stabilise premature neonates improve survival rates, diseases and conditions related to being born preterm are becoming more common.^1^ Unfortunately, the novel technologies and management approaches which lead to good outcomes for premature neonates in high-income countries are often not available in many low-income and middle-income countries (LMICs).^2^ Much international attention is being brought to bear on improving access to life-saving measures for premature neonates, including a goal of mitigating all newborn deaths by 2030.^3–6^ Consequently, there is an increasing focus on managing the complications these neonates can develop as they mature.^7^

One complication of preterm birth is retinopathy of prematurity (ROP), a vasoproliferative disorder of the immature retina. Risk factors include increasing prematurity and exposure to perinatal and postnatal factors.^8 9^ Global estimates in 2010 indicated that there were 14.9 million premature births, 848 300 of whom survived and were at risk of ROP. An estimated 184 700 of those who survived developed any stage of ROP, with 32 200 babies becoming blind or visually impaired.^10^ Rates of blindness from ROP can be higher in LMICs where exposure to modifiable risk factors can be higher, and ROP screening and treatment services are under-resourced or nonexistent.^11^

Screening for ROP, which should include all eligible infants according to local guidelines, starts within weeks of birth.^12^ Unlike screening for other conditions, which usually entails a one-off procedure, screening for ROP may need to be repeated at frequent intervals depending on the clinical findings. If repeat screening is required, it is vital that the findings of earlier screening are immediately available in order to determine whether the condition is regressing, remaining the same or progressing, as this influences the management decision (ie, screen again or treatment is required, or screening can stop). Records of earlier screening can be paper records of the stage, site, signs of plus disease in each eye or retinal images.

Screening is usually undertaken in the neonatal unit; infants no longer inpatients can be screened in an eye department. Binocular indirect ophthalmoscopy by a skilled ophthalmologist is the most common method of ROP screening, but there are several other approaches, all of which have advantages and disadvantages (table 1). A significant advantage of retinal imaging is the capital-labour trade-off it can entail, where the high initial cost of the imaging devices is offset by the lower salary costs of the screeners who do not need to be medically qualified. The ideal situation would be that each neonatal unit has an imaging device so that trained members of the neonatal team or technicians can capture images at a convenient time, with remote image grading by an ROP expert.^13^

**Table 1:** Advantages and disadvantages of different approaches for ROP screening

Technology	Personnel	Main advantages	Disadvantages/Requirements
Indirect ophthalmoscopy	Skilled ophthalmologist.	Immediate diagnosis. Parents can be counselled.	No images to document signs and to use in health education, staff training and to track change over time. Takes ophthalmologists away from other clinical duties.
Wide field imaging	Skilled ophthalmologist takes and grades images at the cot side.	Immediate diagnosis. Parents can be counselled. Provides documentation.	Cost of wide-field imaging systems. Takes ophthalmologists away from other duties.
	Images taken by a trained non-ophthalmologist with image grading remotely by an ROP expert.	Requires less of the ophthalmologist’s time. Provides documentation; screening team can visit multiple NICUs using one device. Artificial intelligence has the potential to guide management decisions.	Cost of wide-field imaging systems. Good internet connection; systems to store and retrieve image. High image quality and robust systems for reporting and communicating management decisions to staff and parent.
	Images taken and initial grading by trained non-ophthalmologists (technicians, nurses) with quality control by an ROP expert.		

NICU, neonatal intensive care unit; ROP, retinopathy of prematurity.

A recent adjunct to screening using indirect ophthalmoscopy is image capture using a smartphone; the light source of the phone is used with a condensing lens, and a device can be used to hold the phone if necessary. Despite the images having a narrow field with a limited view of the retina, they can be used to educate parents and neonatal team members, shared on social media to obtain second opinions and serve as a reference to document the findings.^14^

Where the gold standard for ROP screening, indirect ophthalmoscopy, is not readily available, mainly due to a lack of ophthalmologists able or willing to screen, technology must be designed and tested to improve remote screening capabilities. Target product profiles (TPPs) are used to inform manufacturers of ideal targets and product requirements while aligning with the needs of end users to guide product research and development.^15^ TPPs should include the agreement of many stakeholders on the requirements necessary for a tool to be considered effective for screening.

To address these issues, NEST360 (an international alliance of 17 organisations and 4 governments united to reduce preventable newborn deaths in African hospitals),^16^ the London School of Hygiene & Tropical Medicine and UNICEF led a Delphi-like survey process and consensus meeting with global experts to define the product requirements for an ROP screening device. In this paper, we describe the processes undertaken to develop the TPP, which entailed eliciting the perspectives and opinions of a diverse set of stakeholders.

## Materials and methods

Based on the standardised process established to develop consensus-based TPPs,^17^ we followed six key steps: (1) identified key potential global stakeholders for consultation and input into the TPP development process; (2) isolated the highest priority need for the TPP development through an informal priority-setting exercise with key experts; (3) defined the key TPP domains; (4) hosted an open global webinar featuring global experts and innovative product developers to discuss the challenges with existing technologies as well as opportunities for innovation; (5) completed a Delphi-like survey process with a larger group of key stakeholder to facilitate feedback on the key TPP criteria and (6) held a final consensus-gathering meeting for discussions on criteria where consensus could not be reached in the survey.^18^

### Step 1: identifying key potential global stakeholders

A list of over 370 potential stakeholders (‘potential stakeholder list’) was generated for consultation and input into the TPP development process. The stakeholders were chosen to be representative of implementers and clinicians (including from government, private and non-government organisations), technical agencies and researchers, advocacy organisations/civil society and industry (innovators and manufacturers).

### Step 2: informal priority-setting exercise with key experts

Second, we undertook an informal priority-setting exercise with a select group of key stakeholders to identify the requirements that should be the highest priority in developing the TPP. Given the lack of affordable screening technologies available, a decision was made to focus on a low-cost imaging device for ROP screening.

### Step 3: defining the key TPP domains

Third, the key TPP domains were defined. Product requirements were developed and organised within seven domains: (1) use case (intended use, target operator, target population and target setting); (2) safety and standards (manufacturers’ quality and management system and device regulatory status); (3) technical characteristics (field of view, image resolution, imaging, wavelength, illumination, adjustment of images, dilation needs, time to result for imaging, image output format, system integration, clinical accuracy, accessibility, device weight and patient interface); (4) training and maintenance (user instructions, preventive maintenance and decontamination); (5) power requirements (power source, battery, voltage); (6) durability and lifetime requirements (consumables) and (7) purchasing considerations (price, warranty). These requirements were further described by minimal and optimal product characteristics. Minimal requirements refer to the lowest acceptable or bare minimum requirements which need to be met by all devices. Optimal requirements refer to the ideal targets that products should aim to achieve. The draft TPP had a total of 64 product requirements under the 7 domains.

### Step 4: global webinar

Before distribution of the survey, an open global webinar was held on 22 September 2021. The webinar’s title was ‘Imaging for retinopathy of prematurity (ROP): challenges and innovations’, and it featured leading global experts from Asia, Africa and Latin America, along with innovative product developers. Panellists discussed the challenges with existing technologies and the opportunities for innovation to improve access to reliable, quality ROP screening in resource-limited settings. Over 330 participants registered, 98 of whom attended.

### Step 5: Delphi-like survey process

Following the webinar, the draft TPP developed in step 4 was put into Qualtrics (Qualtrics, Seattle, Washington and Provo, Utah, USA), a commercial survey tool. The link to the Qualtrics survey was sent to those on the potential stakeholder list and those who registered for the webinar. The list included clinicians, implementers, representatives from Ministries of Health, advocacy organisations, international agencies, academic and technical researchers and members of industry. A follow-up reminder email was sent before the deadline on 6 October.

Survey respondents were asked to provide a statement on their level of agreement with each of the proposed product requirements. Agreement was scored on a Likert scale ranging from 1 to 5 (1=disagree, 2=mostly disagree, 3=neither agree nor disagree, 4=mostly agree, 5=fully agree) with an option to opt-out with the selection of ‘other—do not have the expertise to comment’. If participants disagreed with the product requirement (ie, they selected 1, 2 or 3), they were asked to provide an explanation with comments. Participants who agreed with the statements could also provide comments, but these were not explicitly requested. Over 200 comments were reviewed. The percentage agreement was calculated for the minimal and optimal requirements for each product requirement. Agreement was calculated as the ratio of the number of respondents who selected four and five to the total number of respondents. Consensus for the survey product requirements was prespecified at >50% of respondents providing a score of at least 4 on the Likert scale. In total, 36 stakeholders from 17 countries participated in the TPP development process via an online survey. Consensus was achieved for 53 out of 64 (83%) requirements. A classic Delphi process requires at least two rounds of survey ahead of an in-person meeting,^18^ and two survey rounds were planned. However, as 50% consensus was reached for nearly every product requirement after the first round, a second round was not initiated. Thus, a ‘Delphi-like’ method was used.

### Step 6: consensus-gathering meeting

UNICEF and NEST360 convened a virtual consensus-gathering meeting on 10 November 2021. Fifty-six people attended, including the 36 organisations/individuals who participated in the TPP survey (step 5). The purpose of the meeting was to focus on building further consensus on areas where opinions differed. More specifically, product requirements on which fewer than 75% of the respondents agreed or where a distinct subgroup disagreed were discussed. Consensus meeting moderators presented the results and comments from each of the 11 product requirements (17%) with <75% agreement and solicited feedback. Proposed changes to the TPP product requirement were then discussed. This was followed by Zoom polling to assess the consensus level on the proposed changes. Results were exported and analysed in Microsoft Excel. Responders were not required to answer every question, and percentages were calculated based on the response rate to the specific question.

## Results

### TPP survey

The TPP survey engaged leading ROP experts to obtain feedback on a proposed set of product requirements for an ROP screening tool. Over 300 individuals were invited to participate in the Delphi-like survey process, 36 of whom responded (response rate, 12%) from 17 countries on 5 continents. Respondents were clinicians or health professionals (n=26) (72%), product developers or members of industry (n=5) (14%), technicians (n=2) (6%) and other (n=3) (8%).

**Figure 1:**
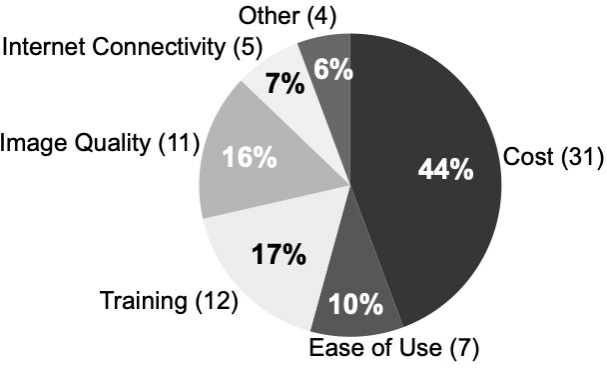
Summary of barriers to access screening for retinopathy of prematurity imaging target product profile.

**Figure 2:**
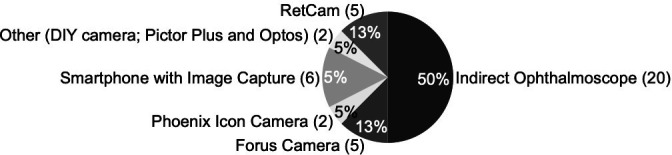
Summary of devices used for screening for retinopathy of prematurity imaging target product profile.

The TPP survey also surveyed respondents on current ROP screening practices and barriers. Twelve of the 36 respondents (33%) reported not currently screening for ROP, saying that the biggest barriers included cost (44%), training (17%) and image quality (16%) (figure 1). Among respondents who were currently screening for ROP, the most common method was indirect ophthalmoscopy (50%). Additional screening tools included smartphones with image capture (15%), RetCam (13%) and Forus Camera (13%) (figure 2).

### Consensus on TPP characteristics

**Figure 3:**
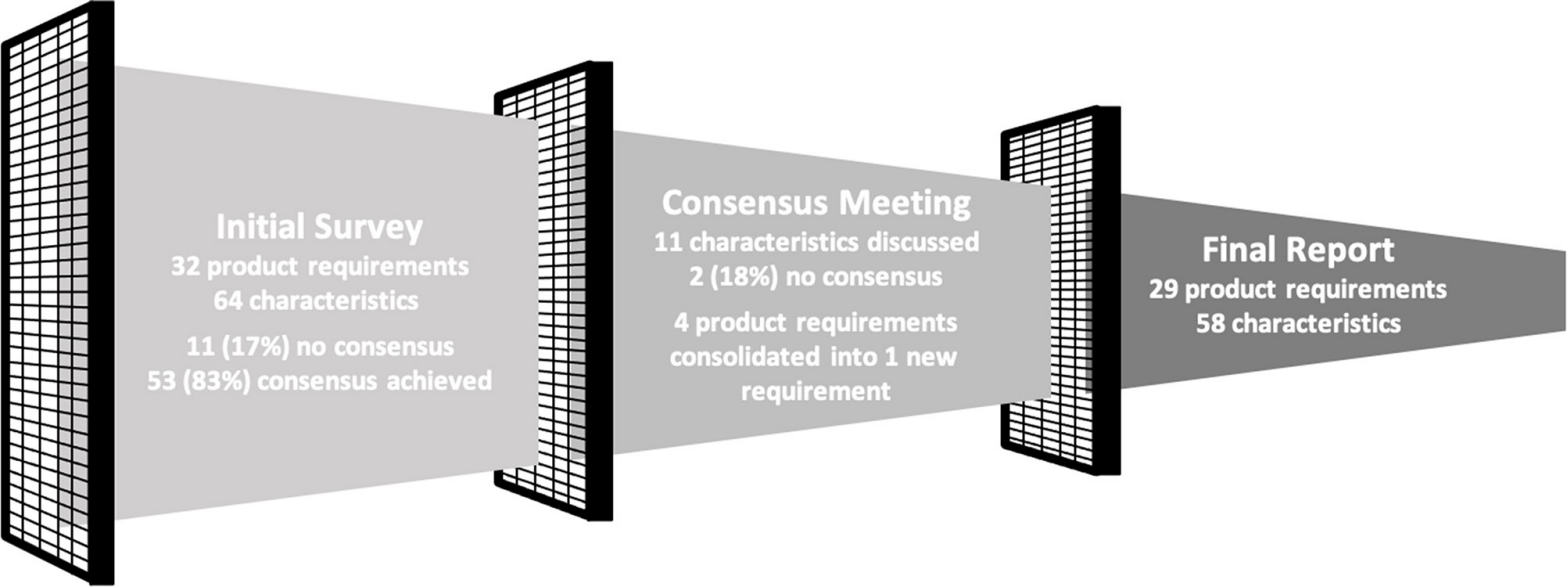
Flow chart summarising consolidation and adaptation of product requirements for final target product profile.

During the survey, consensus was achieved for 53 of the 64 product requirements. The subsequent consensus meeting focused on requirements where fewer than 75% of respondents agreed or where a distinct subgroup disagreed. The 11 product requirements (17%) which were discussed during the consensus meeting are denoted below. All but one of the proposed changes discussed (price) were agreed on (figure 3).

### Target product profile for ROP imaging device

The final product requirements in the TPP are summarised in table 2.

**Table 2:** Final target product profile for an ROP imaging device

	PR	Optimal Refers to the ideal target	Minimal Refers to the lowest acceptable or bare minimum
	Use case		
PR1	Intended use	Screening/Diagnosis of ROP in neonates	
PR2	Target operator	For use in low-income and middle-income countries by a wide variety of healthcare professionals including ophthalmologists, neonatal nurses, clinical officers and technicians and non-clinicians	
PR3	Target population	Neonates (primarily premature and/or low birth weight, at risk for ROP)	
PR4	Target setting	Neonatal units and/or eye department in hospitals in low-resource settings	
	Safety and standards		
PR5	Manufacturers’ quality management system	ISO 13485:2016 medical devices—quality management systems—requirements for regulatory purposes	
PR6	Device regulatory status	At least one of: CE marking, approved by US FDA or another stringent regulatory body of a founding member of International Medical Device Regulators Forum (IMDRF) (eg, Japan or Australia or Canada)	
	Technical characteristics		
PR7	Field of view	170 degrees	36 degrees used with an appropriate condensing lens to be able to view the peripheral retina (into zone 3)
PR8	Image resolution	2048×2048 pixels, 24-bit colours	750×1334 pixels
PR9	Imaging	All three modes available: still, burst mode, video	Still images captured from video
PR10	Wavelength	Colour fundus: white LED (wavelength range 400–750 nm with a peak near 600 nm) Fundus fluorescence angiography: blue LED (dominant wavelength at 480 nm)	
PR11	Illumination	Integrated bright light, 100–6000 lx	
PR12	Adjustment of images	Intensity, gain, balance, brightness, contrast, red free, gamma and focus	Smartphone image editing services
PR13	Dilation needs	No dilation required	Yes, dilation required
PR14	Time to result for imaging	3–4 min	10 min
PR15	Image output format	JPEG, PNG, DICOM, MPEG, HEIC, PACS support	MPEG, HEIC, DICOM
PR16	System integration*	Ability to output to cloud-based support system	Standalone device with ability to output and share images
PR17	Clinical accuracy	100% sensitivity; 99.8% specificity for ROP needing treatment	100% sensitivity; 95% specificity for ROP needing treatment
PR18	Accessibility	Portable: handheld or mobile	Portable
PR19	Device weight (for imaging capture component)	<720 g	No more than 720 g
PR20	Patient interface	Non-contact	Non-invasive contact
	Training and maintenance		
PR21	User instructions	User manual and additional training materials (checklists, videos, guides) in at least one national official language for the country of intended use. Attached to device with labels and markings where possible	User manual provided in at least one national official language
PR22	Preventive maintenance	Preventive maintenance included with additional training materials (checklists, videos, guides)	Manual for preventive maintenance included
PR23	Decontamination	Easy to clean surfaces, compatible with common disinfecting agents	
	Power requirements		
PR24	Power source	Mains with rechargeable battery or solar powered without mains power	Mains power
PR25	Battery	Provides battery backup, autonomy >5 hours, automatic switch to battery in case of power failure, automatic recharge on connection to mains	Battery or power-pack backup
PR26	Voltage	Output spike, surge and transient protection (including lightning), with availability of type I and type II International Electrotechnical Commission (IEC) rating lightning surge protection. Voltage and power input and output metering	Model must match the voltage and frequency of the purchasing country’s local power grid (eg, 110–120 Volts Alternating Current (VAC) at 60 Hz or 220–240 VAC at 50 Hz)
	Durability and lifetime requirements		
PR27	Consumables	None	Pupil dilation drops
	Purchasing considerations		
PR28	Price†	<US$500 ex-works	<US$25 000 ex-works
PR29	Warranty	5 years	1 year

*This is a new product requirement that was developed following the consensus meeting due to the consolidation of various product requirements included in the initial TPP survey. Please refer to the TPP report discussion of PR16: system integration for additional detail and further context.

†There was not 75% voting agreement on both the optimal and minimal product requirement. Please refer to the TPP report discussion of PR28: price for additional detail.

FDA, Food and Drug Administration; PR, product requirement; ROP, retinopathy of prematurity; TPP, target product profile.

### Use case and scope

#### Target operator

The operator was optimally and minimally defined as a wide variety of healthcare professionals, including ophthalmologists, neonatal nurses, clinical officers and paediatricians, technicians and non-clinicians. Given a shortage of paediatric ophthalmologists (for whom devices such as the RetCam are designed) in LMIC settings, the target operator was broadened to include non-clinicians.

### Technical characteristics

#### Field of view

It was agreed that the minimal product requirement should be a field of view of 36 degrees, which can be obtained with an appropriate condensing lens and would enable viewing of the peripheral retina (into zone 3). Optimal requirements indicated a much wider view of 170 degrees. However, there were concerns that smaller fields of view are not ideal for screening and limit usability by non-medical healthcare team members.

#### Image resolution

750×1334 pixels was the minimal resolution agreed on, while 2048×2048 pixels with 24-bit colours was optimal. Commentary included that higher resolution would be of benefit for artificial intelligence algorithms for ROP screening.

#### Imaging

The minimally accepted form of imaging was video with the ability to capture still images, while the optimal form was multimodal, including video, still images and burst modes. More imaging modalities were favourable, time and storage permitting. Potential add-ons of smartphone capabilities and high-resolution modes were also discussed.

#### Dilation needs (discussed at consensus meting)

After considerable discussion at the consensus meeting, the minimal product requirement was that pupil dilation could be used, while the optimal product requirement would strive for a device that would not require pupil dilation. The current standard of care, that is, indirect ophthalmoscopy, requires pupil dilation. However, additional skill, time, and cost are associated with pupil dilation. While a lack of dilation was strongly preferred in low-resource settings, industry members commented that technology with this capacity could not be developed in the foreseeable future, and the closer working distance required could pose new risks to neonates. Agreement could not be reached on the degree of pupil dilation required, so it was excluded from the requirements.

#### Time to result for imaging (discussed at consensus meeting)

The minimally agreed requirement was 10 min for capturing, labelling, saving and adjusting the image, if required. The optimal time was 3–4 min. The discrepancy resulted from variability in the time it can take to capture images, which is highly user dependent. Once captured, current technology such as RetCam, 3Nethra Neo and Optos can deliver results in 2 min or less.

#### System integration (discussed at consensus meeting)

This product requirement was created following the consensus meeting as the result of consolidating four product requirements in the original TPP survey (results output, storage, connectivity and support system). Participants at the consensus meeting advocated that these requirements should not be seen in isolation, as a system is required to store images for future reference and to retrieve specific images from earlier screening episodes.^18^ The device should, at minimum, be a standalone device with the ability to output and share images. Optimally, the device should be able to output to a cloud-based support system.

### Power requirements

#### Battery (discussed at consensus meeting)

The minimally accepted product requirement was that the device would have a battery or power-pack backup for use during power outages. Optimally, the battery backup would provide autonomy for >5 hours, the device would automatically switch to battery when power failed, and would automatically recharge on connection to the mains. A product developer noted that using a battery and power-pack backup could make a product more expensive and heavier.

### Purchasing considerations

#### Price (discussed at consensus meeting)

Consensus (>75% agreement) was not achieved on either the minimal or optimal product requirements of <US$25 000 ex-works minimally and <US$500 ex-works optimally. Cost of device was cited as the most significant barrier to implementing ROP screening. Representatives from Latin America and Africa specifically cited prices >US$2000 as not feasible given local financing models and ROP screening as a comparatively low priority for neonatal units. Cost-sharing models across larger healthcare delivery systems exist but add logistical barriers to access. Manufacturers voiced concern that low prices would compromise the quality of devices, particularly in light of the other requirements discussed.

#### Warranty (discussed at consensus meeting)

A 1-year warranty was the minimum requirement, while a 5-year warranty was optimal. The minimal warranty requirement was accepted as standard practice, but clinicians preferred longer warranties and more favourable terms, which often results in increased costs.

## Discussion

Specific essential product requirements must be met for an imaging device to be an effective tool for ROP screening in resource-limited settings. We report on these key product requirements for scope, safety, performance and technical and operational product requirements in a TPP, which used a consensus-based process with key global stakeholders to define these specifications. The resulting TPP defines the need for an easy-to-use, low-cost imaging device which can be used to screen for ROP in LMICs.

The nature of the consensus-building process informed further considerations for developing a low-cost imaging device for ROP. First, there was a strong emphasis on integrating a screening platform alongside any imaging device. An important distinction was highlighted between imaging (which provides photo documentation) and screening (which requires storage, image retrieval, integration and connectivity into a broader healthcare system). Broader policy measures, along with the development of guidelines, are necessary to catalyse comprehensive clinical management. However, this has broad cost implications for all stakeholders and implies that product developers need to consider longitudinal integration with existing healthcare delivery systems. Furthermore, it was acknowledged that improved screening does not necessarily translate to improved treatment, a separate but related resource-intensive intervention requirement for managing ROP.

Second, the TPP survey highlighted that half of the clinicians (66% of respondents) who were screening for ROP used an indirect ophthalmoscope with pupil dilation. Many non-clinicians were uncomfortable with pupil dilation, a consideration when designing a device for use by a range of non-eye care staff. Only 6% of respondents in the TPP survey self-categorised as ‘technicians’, which was a limitation in the process of developing this TPP. In addition, patients were not consulted for their views.

Finally, cost was both the key barrier and point of disagreement at odds with quality, accessibility and the additional product requirements. As the most significant barrier to care highlighted by our survey, cost will be an important consideration for future discussion and collaboration. The widely varying healthcare economic and policy considerations across countries likely contribute to these discrepancies. A single price may not be effectively implemented across all systems and demographics needing screening. Regional differences in cost requirements are one argument against the one-size-fits-all approach to ROP screening in low-resource settings. Furthermore, not all LMICs are homogenous regarding their progress in developing guidelines and care.

This TPP process was novel in that it successfully brought together diverse stakeholders to reach consensus on the product requirements for an ROP imaging device. The resulting TPP provides a framework which innovators can refer to while developing prototypes for an imaging device for ROP screening. On 24 February 2022 (10 days after the launch), there were 429 downloads of the TPP summary report. Similar processes could also be used to address technological solutions for other eye conditions of public health importance.

## Data Availability

Data are available upon reasonable request.

## References

[1] Bell EF , Hintz SR , Hansen NI , et al . Mortality, in-hospital morbidity, care practices, and two-year outcomes for extremely Preterm infants in the United States, 2013-2018. JAMA 2022;327:248–63. 10.1001/jama.2021.23580 35040888 PMC8767441

[2] Maynard KR , Causey L , Kawaza K , et al . New Technologies for essential newborn care in under-Resourced areas: what is needed and how to deliver it. Paediatr Int Child Health 2015;35:192–205. 10.1179/2046905515Y.0000000034 26053669

[3] Martines J , Paul VK , Bhutta ZA , et al . Neonatal survival: a call for action. Lancet 2005;365:1189–97. 10.1016/S0140-6736(05)71882-1 15794974

[4] Anderson JG , Baer RJ , Partridge JC , et al . Survival and major morbidity of extremely Preterm infants: A population-based study. Pediatrics 2016;138:e20154434. 10.1542/peds.2015-4434 27302979

[5] WHO, UNICEF . Every newborn action plan. 2014. Available: https://www.who.int/initiatives/every-newborn-action-plan

[6] Transforming our world: the 2030 agenda for sustainable development transforming our world: the 2030 agenda for sustainable development preamble. United Nations General Assembly resolution 70/1. 2015. Available: https://undocs.org/en/A/RES/70/1 [Accessed 26 Sep 2022].

[7] Survive and thrive: transforming care for every small and sick newborn. World Health Organization; 2019. Available: https://www.who.int/publications/i/item/9789241515887 [Accessed 26 Sep 2022].

[8] Kim SJ , Port AD , Swan R , et al . Retinopathy of Prematurity: a review of risk factors and their clinical significance. Surv Ophthalmol 2018;63:618–37. 10.1016/j.survophthal.2018.04.002 29679617 PMC6089661

[9] Good WV , Hardy RJ , Dobson V , et al . The incidence and course of retinopathy of Prematurity: findings from the early treatment for retinopathy of Prematurity study. Pediatrics 2005;116:15–23. 10.1542/peds.2004-1413 15995025

[10] Blencowe H , Lawn JE , Vazquez T , et al . Preterm-associated visual impairment and estimates of retinopathy of Prematurity at regional and global levels for 2010. Pediatr Res 2013;74 Suppl 1(Suppl 1):35–49. 10.1038/pr.2013.205 24366462 PMC3873709

[11] Quinn GE . Retinopathy of Prematurity blindness worldwide: phenotypes in the third epidemic. Eye Brain 2016;8:31–6. 10.2147/EB.S94436 28539799 PMC5398741

[12] Kościółek M , Kisielewska W , Ćwiklik-Wierzbowska M , et al . Systematic review of the guidelines for retinopathy of Prematurity. Eur J Ophthalmol 2022. 10.1177/11206721221126286 36120868

[13] Gilbert C , Wormald R , Fielder A , et al . Potential for a paradigm change in the detection of retinopathy of Prematurity requiring treatment. Arch Dis Child Fetal Neonatal Ed 2016;101:F6–9. 10.1136/archdischild-2015-308704 26208954 PMC4717385

[14] Patil J , Patil L , Parachuri N , et al . Smartphone based ROP (S-ROP) screening-opportunities and challenges. Eye (Lond) 2020;34:1512–4. 10.1038/s41433-020-0913-1 32346107 PMC7608357

[15] WHO target product profiles. Available: https://www.who.int/observatories/global-observatory-on-health-research-and-development/analyses-and-syntheses/target-product-profile/who-target-product-profiles [Accessed 26 Sep 2022].

[16] Nest360.Org. n.d. Available: https://nest360.org/

[17] Ivanova Reipold E , Easterbrook P , Trianni A , et al . Optimising diagnosis of Viraemic hepatitis C infection: the development of a target product profile. BMC Infect Dis 2017;17(Suppl 1):707. 10.1186/s12879-017-2770-5 29143620 PMC5688443

[18] Kirby R , Malik A , Gilbert C , et al . Target product profile for retinopathy of Prematurity (ROP) imaging device in low-resource settings. 2022. Available: https://www.unicef.org/supply/media/10896/file/TPP-ROP-imaging-device.pdf [Accessed 26 Sep 2022].

